# Mpox virus nomenclature

**DOI:** 10.1099/jgv.0.002390

**Published:** 2026-11-12

**Authors:** Rachel Fearns, Anthony Griffiths, Carlos Maluquer de Motes, Blair L. Strang, F. Murilo Zerbini

**Affiliations:** 1 Department of Virology, Immunology & Microbiology, Boston University Chobanian & Avedisian School of Medicine, Boston, MA, USA; 2 Department of Molecular Microbiology and Immunology, University of Missouri, Columbia, MO, USA; 3 Section of Virology, School of Biosciences, University of Surrey, Guildford, UK; 4 Department of Medicine, St George’s School of Health and Medical Sciences, City St George’s, University of London, London, UK; 5 Departamento de Fitopatologia/ BIOAGRO, Universidade Federal de Viçosa, Viçosa, Brazil

There can be three names associated with a virus: (i) the name of the disease that it causes, (ii) the common or vernacular name of the virus and (iii) the virus species name. For example, acquired immunodeficiency syndrome (AIDS) is caused by a virus, which is commonly referred to as human immunodeficiency virus-1 (HIV-1), a member of the species *Lentivirus humimdef1*. The World Health Organization (WHO) is responsible for the International Classification of Diseases, which assigns disease names, and the International Committee on Taxonomy of Viruses (ICTV) determines virus species nomenclature. However, the common virus name is not decided by any governing body and is determined by the virology community at large [1].

In 1958, a virus causing a pox-like disease in monkeys in a Danish laboratory was isolated and, accordingly, termed ‘monkeypox virus’ [2, 3]. It is now recognized that this virus is a zoonotic virus that is likely harboured primarily in rodents and that can infect and cause disease in humans [3]. Thus, the name ‘monkeypox virus’ does not reflect the natural host species of the virus. This is also the case for other orthopoxviruses, which are named based on the species from which they were initially identified (e.g. cowpox, camelpox), despite the recognition that these viruses generally use rodents as animal reservoirs in the wild. However, unlike these orthopoxviruses, it has been recognized that the name ‘monkeypox’ has racist connotations and is stigmatizing. Considering these factors, the WHO changed the name of the disease from ‘monkeypox disease’ to ‘mpox disease’ in 2022 [4–6]. A proposal to change the virus species name from ‘*Orthopoxvirus monkeypox’* to ‘*Orthopoxvirus mpox*’ is currently under review by the ICTV [7]. However, there is no clear consensus on the common, vernacular name of the virus as the virology community uses both ‘monkeypox virus’ and ‘mpox virus’.

The *Journal of General Virology* adheres to the ICTV designated names for virus species, updating them as new species names are ratified, and this policy applies to ‘*Orthopoxvirus monkeypox*’. However, with respect to the common, vernacular name, we will henceforth discontinue the use of the name ‘monkeypox virus’ and instead use the name ‘mpox virus’. This will bring the common virus name used by the journal in alignment with the WHO-designated disease name.

## Abbreviations

AIDS: acquired immunodeficiency syndrome
HIV-1: human immunodeficiency virus - 1
ICTV: International Committee on Taxonomy of Viruses
WHO: World Health Organization
